# Evaluation of disinfection of surfaces at an outpatient unit before and after an intervention program

**DOI:** 10.1186/s12879-019-3977-4

**Published:** 2019-04-29

**Authors:** Mara Cristina Ribeiro Furlan, Adriano Menis Ferreira, Larissa da Silva Barcelos, Marcelo Alessandro Rigotti, Alvaro Francisco Lopes de Sousa, Aires Garcia dos Santos Junior, Denise de Andrade, Margarete Teresa Gottardo de Almeida, Mayckel da Silva Barreto

**Affiliations:** 1 University of Mato Grosso do Sul, Três Lagoas, Mato Grosso do Sul Brazil; 2 Ribeirão Preto College of Nursing at University of São Paulo, Ribeirão Preto, São Paulo Brazil; 3 Global Health and Tropical Medicine, Instituto de Higiene e MedicinaTropical (IHMT), Universidade NOVA de Lisboa (UNL), Lisbon, Portugal; 4 School of Medicine of São José do Rio Preto, São José do Rio Preto, São Paulo Brazil; 5 Faculty of Philosophy, Sciences and Letters of Mandaguari, Mandaguari, Paraná Brazil; 6 Campus Universitário - Bairro Monte Alegre, Avenida dos Bandeirantes, 3900, Ribeirão Preto, CEP: 14040-902 SP Brasil

**Keywords:** Disinfection, Hospital housekeeping, Health education, Infection control, Ambulatory care

## Abstract

**Background:**

Cleaning and disinfection processes must be improved so that there is a reduction in environmental contamination of frequent-contact surfaces. The objective of this study was to evaluate cleaning and disinfection of surfaces at a specialized healthcare unit after an intervention program.

**Methods:**

Exploratory, longitudinal, and correlational study carried out in a medium-complexity clinic. Two hundred and forty samples from five surfaces were collected during three phases: diagnosis; implementation of an intervention program; and evaluation of immediate and long-term effects. In total, 720 evaluations were made, performed through three monitoring methods: visual inspection; adenosine triphosphate bioluminescence assay (ATP); and aerobic colony count (ACC). The Wilcoxon, Mann-Whitney, and Fisher’s Exact tests were run to analyze data statistically.

**Results:**

Cleaning and disinfection of surfaces were not being performed properly in most cases. Failure rates of surfaces reached 37.5 and 100% when the ATP and ACC procedures were used, respectively. However, after an intervention program, an improvement occurred. Success rates increased by 43.96% (ATP) and 12.46% (ACC) in phase I, by 70.6% (ATP) and 82.3% (ACC) immediately after interventions, and by 76.52% (ATP) and 85.76% (ACC) two months after the changes, showing that the program was effective.

**Conclusion:**

The present study reveals that implementing intervention actions with a cleaning and healthcare team brings benefits to prevent the spread of pathogenic agents through frequently touched hospital surfaces.

## Background

Ensuring clean and safe environments is an essential component of effective health care, which is fundamental to prevent and control healthcare-associated infections (HAIs). Annually, these infections affect hundreds of millions of people worldwide and lead to significant mortality rates and financial losses for health systems [1].

Healthcare-associated infection rates remain high, among other reasons, because surfaces at healthcare services are not sanitized according to standardized institutional protocols. In some cases, these protocols do not even exist. Consequently, cleaning and disinfection processes must be improved so that there is a reduction in environmental contamination of frequent-contact surfaces, thus minimizing HAI rates and harmful consequences to patients, professional teams, health systems, and society at large [2].

Cleaning quality depends on specific protocols for each healthcare facility, enough professionals to implement these protocols, and a practical monitoring process for. Actions carried out in hospitals in Germany [3], Taiwan [4], and the United States [5] showed an increase of 69, 44.2, and 34% in clean surfaces, respectively. Although these results are promising, they are restricted to hospital settings.

The literature indicates a gap regarding the evaluation of cleaning and disinfection of surfaces in outpatient units. It is necessary to stress that regulatory agencies have been emphasizing the importance of preventing and controlling HAIs in healthcare environments other than hospital settings.

Given the need to examine surface cleaning and disinfection in outpatient contexts and to implement actions to improve them, the objective of the present study was to assess cleaning and disinfection of surfaces, in a healthcare unit which offers specialized care, before and after an intervention program.

## Methods

This was an exploratory, longitudinal, and correlational study carried out in a public outpatient clinic that assists 100,000 adult and older people in the center-west of Brazil. The services provided include medical specialties, endoscopy, outpatient surgeries, treatment of chronic lesions, and imaging tests. It has a mean annual delivery of 9200 appointments and 6060 procedures. The operation schedule is from Mondays to Fridays, from 6 a.m. to 5 p.m., and the population assisted is referred by primary health care units.

The institution has 19 professionals, among whom six worked in the nursing team and two belonged to the cleaning staff. The nursing professionals performed the routine cleaning of the furniture and equipment in the rooms where procedures were carried out, whereas the cleaning staff performed the terminal cleaning of these rooms and routine cleaning of other rooms in the outpatient clinic.

Samples were collected from five selected surfaces, from July to December 2015 (winter and spring) according to the frequency with which they were touched by users and professionals [6]. The objects were: a bandage trolley; a stretcher; a reception desk; a Mayo table; and an operating table. The mattress of the stretcher was made of polyvinyl chloride covered with knitted polyester and polypropylene, the reception desk of marble, and the other surfaces of stainless steel.

The disinfectant used for cleaning and disinfection of the surfaces was Incidin® Extra N at 5% (Ecolab Deutschland GmbH), made up of alkyl dimethyl benzyl ammonium chloride, nonionic surfactant, glucoprotamin, solvent, complexing agents, an anticorrosive agent, and water. The product does not require rinsing and it is indicated to the disinfection process of fixed surfaces.

Both the nursing team and the cleaning staff used sprays to clean and disinfect the studied surfaces, with a note that was printed and attached to them regarding its expiration within seven days after the product was diluted. For the cleaning and disinfection process the cleaning staff used 100% cotton cloth, and the nursing team used simple paper towels (100% cellulose fibers). The cleaning movement was unidirectional. Regarding the frequency of cleaning and disinfection of the surfaces, they were carried out in the end of each patient’s procedure for the stretcher of the dressing room, and in the end of each shift (morning and afternoon) for other surfaces.

The study was performed in three phases and sampling occurred twice a week. Collection days were determined randomly. Phase I (diagnosis, no intervention), lasted one month and had the objective to evaluate the efficiency of surface cleaning and disinfection procedures performed in the unit routinely. At this stage, the team was not informed about the real goal of the study or regarding the surfaces that would be assessed. This choice was intended to avoid the *Hawthorne* effect [6]. When employees asked questions about the presence of researchers during data collection, they were informed that the cleaning products used in the unit were being evaluated [7].

In Phase II (intervention), data collected during Phase I were used to formulate an intervention program and give the cleaning staff (*n* = 2) and the nursing team (*n* = 6) feedback about the cleaning procedures, given that they were the professionals responsible for cleaning and disinfection of the mentioned surfaces. Cleaning the reception desk was the cleaning staff’s duty, and the other surfaces were sanitized by the nursing team.

The intervention program consisted of three steps. The first consisted of the development of two sections, lasting one hour, with a dialogue-based expositive class addressing content related to the following topics: principles of infection prevention and control; basic microbiology; hand hygiene; relevance of surfaces in the pathogen chain of transmission; guidance on surface cleaning and disinfection techniques; and correct dilution and handling of hygiene products.

In the second step, the results obtained in Phase I were presented, including showing employees the culture plates with colony-forming units (CFUs) and the outcomes of monitoring through visual inspection, ATP, and ACC assays produced in Phase I. Seeing the culture plates, the professionals were surprised, because despite being visually clean many surfaces presented CFU levels higher than the advocated limit.

The third step consisted of the standardization of surface cleaning and disinfection practices, such as cleaning routines and the use of microfiber cloths (80% viscose, 15% polypropylene, and 5% polyester). It was decided that microfiber cloths should be folded into four parts, every side should be used, and replacement would happen whenever necessary [7].

Use of the product Incidin Extra N 0.5% (Ecolab Deutschland GmbH), was maintained. It was to be sprayed on the microfiber cloths until a proper dampness was reached without soaking the cloths with an excessive volume of liquid [6–8].

The present investigation did not establish a directional flux for cleaning movements (circular, unidirectional) to be followed, in accordance with a recent study [9]. Regarding friction, professionals were oriented to apply an adequate force for at least 15 s [6–9].

Afterward, a protocol addressing surface cleaning and disinfection for the institution was designed and a practical class was prepared to teach the application of the technique. The first demonstration was carried out by a researcher, and subsequently the staff members were asked to execute the protocol, which allowed for adjustment of practices that were still inadequate. There were two meetings to train the teams, each lasting one hour.

Still in Phase II, following the steps concerning the intervention program mentioned previously there was an evaluation of the examined surfaces, a procedure that spanned four weeks. Data collection was based on the same actions developed in Phase I (diagnosis). Phase II also included providing feedback about the results and additional guidance, according to the requests of the professionals [6–8].

Phase III (washout period) began two months after the end of Phase II and lasted for four weeks, during which the same actions from Phase I were applied. This step allowed evaluation of whether the practices were maintained over time with no new intervention, which permitted verifying whether the implemented measures were incorporated into professionals’ practice.

Seven hundred and twenty samples/evaluations were collected/examined in the investigation (240 in each phase), and 80 samples were assessed through a specific method (visual inspection, ATP bioluminescence, and ACC) at each step. First, visual inspection was performed first, then ATP and CCA samples were collected in the sequence, being one beside the other, for each surface per collection day. Surfaces were sampled by the main researcher only, immediately before and 10 min after the end of the morning or afternoon cleaning session, depending on the surface that would be examined in a certain period.

This procedure allowed the objects to dry completely and reduced the chances of wrong outcomes in ATP bioluminescence and ACC assays caused by contact between cleaning products and reagents [6]. In addition, researchers made sure to carry out the experiments as quickly as possible after cleaning of surfaces to prevent recontamination [9, 10].

Visual inspection was executed by considering dirty or reproved any surface presenting at least one of the following items: dust; liquid; waste (organic matter or not); scratches; cracks; paint stripping; and paint or glue stains [6].

The ATP bioluminescence assay was used to assess microscopic organic matter, with a portable luminometer (Clean-Trace™ ATP System, 3 M Company, St. Paul, MN) and swabs (3 M™ Clean-Trace™ Surface ATP Test Swab). Collection was carried out according to the instructions provided by the manufacturer. Surfaces were considered clean or approved when the output of the device was lower than 250 relative light units (RLUs) [10, 11] in a 100 cm^2^ area.

To monitor the quantity of total aerobic microorganisms, Rodac® Plate (replicate organism detection and counting) contact plates (24 cm^2^) were used. These consisted of trypticase soy agar and neutralizers for the disinfectant used. The plates were pressed on the evaluated surfaces for 10 s in places adjacent to those where samples for ATP bioluminescence assay were collected, and subsequently inserted into an incubator at 37 °C for 24 to 48 h [12–14].

Plate reading was performed with a digital colony counter (Logen LS6000, Texas Instruments Inc., Dallas, TX). The threshold adopted for surfaces to be considered clean or approved was less than 2.5 CFUs/cm^2^, that is, less than 60 CFUs/plate [10, 14].

Data analysis required the application of the following statistical tests: the Wilcoxon test, to compare quantification results by ATP and ACC assays before and after cleaning in each examined surface and study phase; the Mann-Whitney test, to parallel the difference in microbial counting and quantification through ATP bioluminescence on each examined surface and in each study phase; and the Fisher exact test, for two proportions, in order to assess variations in the outcomes of visual inspection. All of the statistical tests had a 5% level of significance, or *p* < 0.05, and the software used was Minitab 17 (Minitab Inc.) and MedCalc 16.8 (MedCalc®).

Differences in the outcomes of the ACC and ATP bioluminescence assays were compared to verify the existence of significant variations between the applied methods and calculated according to the formula:

$$ Variation\%\left( ATP\  or\  CFU\right)={\frac{after- before}{before}}^{\ast }100 $$

Positive variations indicate that data collected after intervention had higher values in comparison with those obtained before intervention. The opposite stands for negative variations. Consequently, positive differences point to an increase in the number of RLUs or CFUs, and negative differences reveal a reduction in these outcomes.

## Results

The comparison of the results between the pre- and post-intervention situations of the five studied surfaces is shown in Table 1. Phase I (diagnosis) revealed the existence of significant differences between RLU scores before and after cleaning for two surfaces only, the operating table (*p* = 0.030) and the Mayo table (*p* = 0.014). Regarding the microbial count, only one surface presented a significant difference, the operating table (*p* = 0.021). In addition, ATP bioluminescence and ACC outcome differences reached 37.5 and 100% respectively for some surfaces, which caused them to fail. This fact shows that surface cleaning and disinfection was not being carried out effectively most of the time.

**Table 1:** Medians (minimum value; maximum value) referring to Phases I, II and III of the samples obtained from the surfaces

Phase I (no intervention)											
Analysis method	Cleaning	Reception desk	*p* value	Bandage trolley	*p* value	Stretcher	*p* value	Operating table	*p* value	Mayo table	*p* value
ATP (RLU)1	Before	209 (71;1365)	0.834	124 (60;299)	0.107	304 (156;1452)	0.624	564 (150;2417)	**0.030**	324.5 (120;735)	**0.014**
	After	203 (34;575)		69 (13;338)		189 (58;2083)		207 (33;1415)		42.5 (11;62)	
Bacteria (CFU/cm^2^)1	Before	96 (53;219)	0.183	40.5 (4;96)	0.107	78.5 (61;197)	0.441	113.5 (37;300)	**0.021**	87.5 (64;154)	0.624
	After	63.5 (31;153)		45.5 (37;94)		53 (6;176)		66.5 (2;112)		50.5 (3;172)	
Variation analysis2	RLU	−2.3 (−91.8;349.2)	0.563	−49.5 (−85.3;52.3)	**0.018**	−19 (−94.9;410.5)	0.713	−60.8 (− 93.5;19.8)	0.494	−89 (− 93.6;-76.9)	**0.031**
	CFU	−31.3 (−71.2;88.9)		71 (−39;825)		−32 (−96.8;100)		−26.5 (−99.2;10.6)		−44 (−96.4;100)	
Phase II (short-term evaluation)											
ATP (RLU)1	Before	239 (96;958)	0.183	114 (70;533)	**0.014**	295 (59;886)	**0.014**	166 (50;1515)	**0.021**	194.5 (41;256)	**0.014**
	After	61.5 (40;326)		36.5 (17;95)		41.5 (25;483)		44.5 (11;90)		44.5 (17;83)	
Bacteria (CFU/cm^2^)1	Before	31.5 (7;300)	**0.042**	58 (6;300)	**0.014**	47 (6;257)	0.183	17.5 (2;62)	**0.014**	59.5 (26;173)	**0.014**
	After	11.5 (2;18)		6 (0;30)		5 (0;28)		2 (0;17)		2.5 (0;31)	
Variation analysis2	RLU	−72 (−86;193.7)	0.792	−72.4 (−90.8;-56.6)	0.083	− 67.6 (−93.8;-36.5)	0.189	−69.5 (−97.7;1.6)	0.462	−71.9 (−87.9;-8.1)	**0.010**
	CFU	−65.1 (−99.3;57.1)		−87.0 (−100;-16.7)		−92.3 (−100;283.3)		−76.3 (−100;-50)		−93.8 (− 100;-50.8)	
Phase III (after intervention – long-term evaluation)											
ATP (RLU)1	Before	633 (109;1327)	**0.014**	237.5 (53;530)	**0.014**	451 (113;3809)	**0.014**	1053 (140;9597)	**0.014**	109 (23;1989)	0.183
	After	123.5 (16;792)		41.5 (15;94)		56.5 (22;166)		95 (59;610)		46.5 (17;91)	
Bacteria (CFU/cm^2^)1	Before	72.5 (9;294)	0.234	32.5 (11;251)	0.183	45 (3;179)	0.141	15 (3;252)	0.353	13.5 (0;39)	**0.036**
	After	14.5 (3;60)		4.5 (2;39)		4 (0;6)		2.5 (0;178)		1 (0;14)	
Variation analysis2	RLU	−78.4 (−95.6;-12.7)	0.833	−82.4 (−93;-30.2)	0.713	−87.1 (−95.6;-58.4)	0.636	−85 (−99.4;-34.3)	0.713	−52.1 (−99.1;78.3)	0.271
	CFU	− 84.2 (−96.3;122.2)		−88.5 (− 99.2;105.3)		−89.2 (−100;100)		−72 (−100;1680)		−94.9 (−100;0)	

Note: *CFU* colony-forming unit, *ATP* adenosine triphosphate, *RLU* relative light unit. 1*p* value referring to the Wilcoxon rank-sum test with *p* < 0.05. 2*p* value referring to the Mann-Whitney test with *p* < 0.05

Values captured in bold are statistically significant

Analysis of ATP bioluminescence (denoted by RLU) and ACC (symbolized by CFU) results of Phase I uncovered significant differences in the bandage trolley (*p* = 0.018) and the Mayo table (*p* = 0.031). In both objects, the variation of RLU outputs had a higher magnitude than the CFU reading. That is, the decrease in RLUs after cleaning was significantly superior to the reduction of microbial count on both surfaces.

Phase II presented several cases of statistically significant differences, which suggests that the intervention was effective. There were four cases for the ATP bioluminescence technique: (bandage trolley (*p* = 0.014); stretcher (*p* = 0.014); operating table (*p* = 0.021); and Mayo table (*p* = 0.014)), four for the microbial counting method (reception desk [*p* = 0.042], bandage trolley [*p* = 0.014], operating table [*p* = 0.014], and Mayo table [*p* = 0.014]) and only one case involving a comparison between ATP bioluminescence and ACC (Mayo table [*p* = 0.010]). In all of these cases, the outputs obtained through ATP bioluminescence and ACC were lower after cleaning and disinfection. That is, these procedures had a positive impact on reducing contamination. This effect was considered significant for the mentioned surfaces.

In Phase III, the reception desk (*p* = 0.014), bandage trolley (*p* = 0.014), stretcher (*p* = 0.014), and operating table (*p* = 0.021) exhibited a decreased contamination level as measured by ATP bioluminescence, and the Mayo table (*p* = 0.036) presented a lower CFU output. Additionally, RLU results were lower after cleaning for every surface examined. Therefore, the intervention effect could be detected even in the long run.

After the intervention program, the percentage of post-cleaning and disinfection approval of the surfaces grew by 43.96% (ATP) and 12.46% (ACC) in Phase I, by 70.6% (ATP) and 82.3% (ACC) immediately after interventions, and by 76.52% (ATP) and 85.76% (ACC) two months after implementation, evidence that the program produced results.

As for visual inspection, data showed that in Phases I and III, regardless of cleaning, the bandage trolley, and the Mayo table were not approved. The other surfaces were approved in all evaluations before and after cleaning and disinfection in Phase I.

Still, according to visual inspection in Phase III, the reception desk was the surface which presented the highest approval percentages before and after cleaning (100%). For the operating table, the increase in the approval was significant, showing a positive effect on this surface (*p* = 0.026). The approval index before cleaning was 37.5% and increased to 100% after the measures.

In Phase III, the reception desk remained the cleanest surface according to visual inspection (100%), followed by the stretcher (75%) and the operating Table (50%). The bandage trolley had a slight improvement in approval (12.5%). The Mayo table failure index remained high in this phase, as a consequence of paint deterioration in most cases. The proportions of approved and unimproved surfaces were similar (*p* > 0.05) (Table 1).

**Fig. 1:**
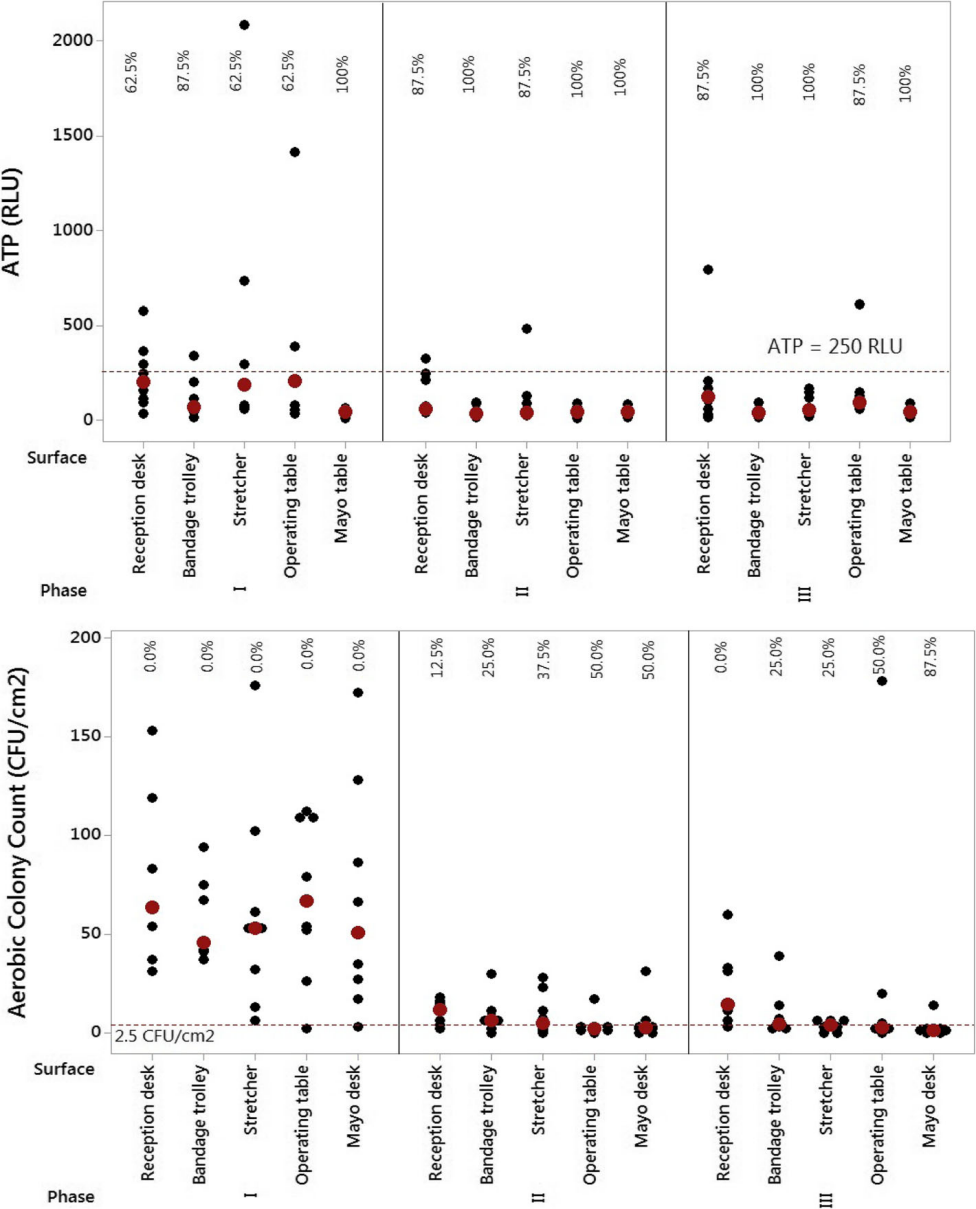
RLU and CFU values for the five examined surfaces in the three phases of the study. Note: Percentages refer to approval rates. Black points indicate individual RLU and CFU values and red points designate the medians of the distributions

Figure 1 displays the evolution of each RLU and CFU output per evaluated surface and phase. Approval was associated with values lower than 250 RLUs and 2.5 CFUs/cm^2^. Figure 1 exhibits individual values for ATP bioluminescence indexes for the five surfaces in the three phases after intervention.

Data in Fig. 1 reveal that all of the surfaces presented approval indexes higher than 50% in every phase when evaluated through the ATP bioluminescence method. It is noteworthy that surfaces achieved high approval rates in Phases II and III in comparison with Phase I. The Mayo table was the only surface that had 100% approval regarding the ATP limit (250 RLUs) in every phase.

Analysis of surfaces through the ACC method showed evidence that the intervention had a positive effect on the percentage of approved surfaces, taking into account a cutoff of 2.5 CFUs/cm^2^. In Phase I, all of the surfaces were improved, but the approval percentage increased after intervention, reaching 50% of approval for the operating and Mayo tables in Phase II and 87.5% of approval for the Mayo table in Phase III.

## Discussion

Cleaning and disinfection of outpatient health facilities may be improved by implementing an intervention program oriented toward that goal. The findings of the present study revealed that, even in a medium-complexity health institution, where cleaning and disinfection were not being carried out properly and led to high RLU and CFU outputs, specific and scheduled interventions are effective.

The growing concern with the risk to contract HAIs in outpatient healthcare services has been posing a challenge to caretakers, professionals, and researchers worldwide, despite the existence of literature informing about the severity of this scenario. A study that evaluated over 5000 outpatient clinics in the United States revealed that 18.8% of these facilities did not have a correctly performed cleaning procedure on frequently touched surfaces in patient care areas [15]. A study carried out in five primary healthcare units in Portuguese municipalities identified major flaws in the cleaning and disinfection process by applying visual inspection and ATP bioluminescence [16].

Consequently, it is essential that evaluation of surface cleaning and disinfection be performed, because it provides valuable information to develop surveillance programs that rate cleaning and disinfection protocols for outpatient clinical settings. Interventions with healthcare and cleaning staff seem to be a strategy that helps achieve better results in the cleanliness of facilities.

Investigations that assessed intervention programs to improve surface cleaning and disinfection in tertiary health care (clinical medicine) [8] and secondary health care (urgency and emergency) [6] showed that there was a significant improvement after interventions, with a reduction in the number of surfaces needing reassessment according to ATP bioluminescence and microbiological culture experiments. However, the effect of these interventions was not maintained on most surfaces or in their totality, resulting in an increase in CFU and/or RLU outputs after two months [6, 8]. The main factors that explain this outcome are lack of monitoring, lack of observation of cleaning practices, and lack of supervision and guidance, stressing the need for continuity of intervention strategies to train teams.

Alternatively, a study carried out in a primary healthcare unit observed a decrease in microbial charge through ACC and ATP bioluminescence on five surfaces by comparing outcomes obtained before and after the implementation of a cleaning and disinfection program. This effect persisted for two months, when a new assessment was performed using the same techniques [7].

Several aspects may be related to negative impacts of interventions and help explain these results. The main ones are nonadherence to the protocol; inadequacy of the executed procedures; use of contaminated materials, utensils or equipment; and lack of feedback to the team [7, 16]. The latter has been pointed out as a decisive element to achieve positive results, given that it tends to encourage the team, allowing the team to raise awareness of its role in behavior change.

It must be taken into account that, in Brazil, in the same way as in other developing countries, there is a lack of investment in prevention, control, and surveillance in outpatient settings. In addition, units and professionals that belong to this health sphere have limitations to their ability to prevent and control the occurrence of infections in this type of environment, given that the care dynamics differ significantly from those existing in hospitals [17].

The authors assume that, in the examined unit, commitment and working conditions may have contributed to maintain the improvement of surface cleaning and disinfection, even in the long run. There are many factors related to the positive performance of an intervention oriented toward surface cleaning and disinfection monitoring, such as measuring ATP bioluminescence. However, involvement and interest in the improvement of surface cleaning processes is determined in terms of its efficacy [7, 12].

It is important to emphasize that during Phase II (intervention), several questions were asked by the cleaning staff and the nursing team, and visualization of colonies on culture plates resulted in a positive effect by surprising the participants. The examined surfaces were often clean according to visual inspection. This practice allowed employees to realize that they really needed to change the way they carried out cleaning and disinfection to prevent contamination by microorganisms and eventually decrease the chances of infection.

The surface cleaning evaluation methods used in the present study are educational tools, and sometimes process tools, for professionals involved directly and indirectly in the activities. Nevertheless, they are not enough to characterize the cleanliness of a healthcare institution, because only a limited part of a larger area is evaluated through these methods.

Therefore, the authors believe that visual inspection, ATP bioluminescence, and microbiological counting should be used with caution to evaluate surface cleaning and detect neglected areas. It is advisable that a member of the nursing team becomes responsible for controlling cleaning and disinfection of the area, and provides employees involved in the cleaning process with feedback.

The results corroborated a review [18] suggesting that monitoring with ATP bioluminescence is the most appropriate to identify which surfaces must be cleaned, whereas microbiological counting is ideal to identify whether the surfaces were cleaned properly. Figure 1 shows that the percentage of approved surfaces in all phases of the study is higher when they are assessed through ATP bioluminescence rather than microbiological counting.

The present study has some limitations. It was carried out in only one healthcare institution, which may limit generalization of results. It was not possible to determine whether the microorganism colonies found through the ACC method could cause infection. In addition, because practice monitoring was performed by healthcare professionals, it is necessary to consider the possibility that the Hawthorne effect may have influenced the outcomes. In contrast, one of its strengths was the fact that all sample analyses and evaluations were carried out by the same researcher, which increased the chances of a standardized performance.

## Conclusion

The actions taken in an intervention program for surface cleaning and disinfection with a cleaning staff and a nursing team had a positive effect on the cleaning efficiency of an outpatient facility. This impact was both immediate and long-term (two months after the implementation of the program).

The present study provides important information about the prevalence of CFUs and ATP on surfaces in an outpatient setting. It showed that actions in the field of cleaning and disinfection with the cleaning staff and nursing team are effective and help prevent dissemination of microorganisms on frequently touched medical surfaces.

The authors suggest that new investigations be designed, with different configurations of outpatient clinics, to monitor cleaning and disinfection, so that it is possible to compare situations and obtain a basis for regular surveillance and adoption of protocols. The need for future studies in this area is especially compelling given the sparse literature on this topic.

## Abbreviations

ACC: Aerobic colony count
ATP: Adenosine triphosphate
CFUs: Colony-Forming Units
HAI: Healthcare-associated infections
RLU: Relative light units
